# Comparative efficacy and safety of antidiabetic agents in Alzheimer's disease: A network meta-analysis of randomized controlled trials

**DOI:** 10.1016/j.tjpad.2025.100111

**Published:** 2025-02-28

**Authors:** Zixin Cai, Jiaxin Zhong, Guanghui Zhu, Jingjing Zhang

**Affiliations:** a National Clinical Research Center for Metabolic Diseases, Key Laboratory of Cardiometabolic Medicine of Hunan Province, Metabolic Syndrome Research Center, The Second Xiangya Hospital of Central South University, Changsha, Hunan, PR China; b The Affiliated Children's Hospital of Xiangya Medical School, Central South University (Hunan Children's Hospital), Hunan Provincial Key Laboratory of Pediatric Orthopedics, Changsha, Hunan, China; c Furong Laboratory, Changsha, Hunan, China; d MOE Key Laboratory of Rare Pediatric Diseases, University of South China, Hengyang, Hunan 421001, PR China; e School of Pediatrics, University of South China, Changsha, Hunan 410007, PR China

**Keywords:** Alzheimer's disease, Antidiabetic agents, Cognitive performance, Amyloid-β deposition, Network meta-analysis

## Abstract

**Background:**

Alzheimer's disease (AD) is a progressive neurodegenerative disorder with limited treatment options. Emerging evidence suggests that antidiabetic agents may offer neuroprotective effects by targeting shared pathophysiological mechanisms such as insulin resistance and neuroinflammation. However, the comparative efficacy, and safety of these agents in the treatment of AD remain unclear.

**Objectives:**

This study aimed to systematically evaluate and compare the efficacy and safety of antidiabetic agents for improving cognitive outcomes, reducing amyloid-β (Aβ) deposition, and managing adverse effects in patients with AD, using a network meta-analysis of randomized controlled trials (RCTs).

**Methods:**

A comprehensive literature search was conducted across multiple databases to identify RCTs examining the effects of antidiabetic agents in patients with AD. The primary outcomes included cognitive performance (e.g., MMSE scores), Aβ deposition (measured via CSF biomarkers), and safety/adverse effects. A network meta-analysis was performed to integrate direct and indirect evidence, ranking interventions using Surface Under the Cumulative Ranking (SUCRA) probabilities. Risk of bias was assessed using the Cochrane risk-of-bias tool.

**Results:**

A total of 26 studies, involving 7,361 participants, were included in the analysis. The interventions evaluated included insulin detemir (both low-dose and high-dose), liraglutide, exenatide, metformin, and pioglitazone. Both low-dose insulin detemir (mean difference: 2.10, 95 % CI: 1.04 to 3.15), high-dose insulin detemir (mean difference: 1.40, 95 % CI: -0.07 to 2.88), exenatide (mean difference: 1.19, 95 % CI: 0.06 to 2.32), and metformin combined with exenatide (mean difference: 1.06, 95 % CI: -1.68 to 3.80) showed cognitive improvements compared to placebo. Among these, low-dose insulin detemir demonstrated the most significant improvement. In terms of reducing Aβ deposition, metformin ranked highest in effectiveness, with the highest SUCRA score (84.6), followed by high-dose insulin detemir (SUCRA: 54.1). Low-dose insulin detemir (SUCRA: 51.1) also demonstrated moderate efficacy. Low-dose insulin detemir showed some reduction in Aβ deposition (mean difference: -0.31, 95 % CI: -2.82 to 2.20), although statistical significance was limited. Liraglutide exhibited the highest rate of study treatment withdrawal (mean difference: 1.97, 95 % CI: -0.07 to 4.00), while pioglitazone demonstrated the lowest withdrawal rates (mean difference: 0.07, 95 % CI: -0.03 to 0.17).

**Conclusions:**

This network meta-analysis provides valuable insights into the comparative efficacy and safety of antidiabetic agents in AD. Low-dose insulin detemir demonstrated the most significant cognitive improvement and a moderate effect on reducing Aβ deposition. Metformin emerged as the most effective agent for reducing Aβ levels, though its effects on cognitive function were less pronounced. Safety profiles varied, with liraglutide associated with the highest rate of treatment withdrawals, while pioglitazone demonstrated the lowest incidence of treatment-related discontinuations. These findings support the potential use of antidiabetic agents, particularly insulin detemir, as a therapeutic option for AD, although further studies are needed to confirm their long-term benefits and safety.

## 1 Introduction

Alzheimer's disease (AD) is a progressive neurodegenerative disorder and the leading cause of dementia globally [1]. Characterized by a gradual decline in cognitive functions such as memory, language, and executive abilities, AD ultimately results in severe functional impairment. The pathophysiology of AD is multifactorial and complex, involving processes such as amyloid-β (Aβ) aggregation, tau protein hyperphosphorylation, neuroinflammation, and synaptic dysfunction [2]. Recently, brain insulin resistance has emerged as a significant factor in AD progression, with some researchers referring to AD as "type 3 diabetes" [3]. Despite substantial advances in understanding the molecular mechanisms underlying AD, current pharmacological treatments—primarily cholinesterase inhibitors and NMDA receptor antagonists—are limited to symptomatic relief and do not address the disease's root causes [4]. This gap in effective treatment has underscored the urgent need for novel therapeutic approaches that target the underlying pathophysiological mechanisms of AD.

A growing body of evidence suggests that the pathophysiological mechanisms of AD overlap significantly with those of type 2 diabetes mellitus (T2DM) [5]. Both diseases involve insulin resistance, chronic inflammation, oxidative stress, and vascular dysfunction. Notably, insulin resistance in the brain is implicated in impaired glucose metabolism, synaptic dysfunction, and the exacerbation of Aβ and tau pathologies [6]. These shared mechanisms have led to increasing interest in the repurposing of antidiabetic agents as potential therapeutic candidates for AD. By targeting insulin signaling pathways, enhancing glucose metabolism, and exerting anti-inflammatory effects, antidiabetic drugs—such as GLP-1 receptor agonists, insulin analogs, and thiazolidinediones—offer promising neuroprotective properties. Given their potential to mitigate both cognitive decline and neuropathological changes associated with AD, these agents warrant further systematic investigation.

Preclinical and clinical studies have increasingly focused on the potential of antidiabetic agents to modify AD pathology. In animal models, insulin analogs and GLP-1 receptor agonists have demonstrated the ability to improve synaptic plasticity, enhance glucose metabolism, and reduce Aβ deposition [7]. GLP-1 receptor agonists, initially developed for the treatment of T2DM, have shown neuroprotective effects in models of neurodegenerative diseases. These compounds cross the blood-brain barrier, improve memory formation and synaptic plasticity, and reduce neuroinflammation, oxidative stress, and amyloid plaque load in AD models [8]. Similar protective effects have been observed in Parkinson's disease models, where GLP-1 receptor agonists help protect dopaminergic neurons and preserve motor control [8]. Clinical trials further support the potential of antidiabetic agents in AD treatment. For instance, metformin, a first-line treatment for T2DM, has shown promise in enhancing neuronal glucose uptake and improving global cognitive outcomes in patients with mild cognitive impairment and AD [9]. Pioglitazone, a PPAR-γ agonist, has demonstrated the ability to modulate microglial activity and reduce neuroinflammation, although its effects on cognition have been inconsistent across studies [10]. While these findings are promising, the effects of antidiabetic drugs on AD biomarkers and clinical outcomes remain under active investigation.

Despite these encouraging results, the therapeutic potential of antidiabetic agents for AD remains inconsistent across studies. Not all trials have shown significant cognitive improvements or reductions in AD biomarkers, and the efficacy of certain agents may depend on factors such as disease stage, dosage, and route of administration [11]. Furthermore, the safety and tolerability profiles of these drugs vary; while metformin is generally well-tolerated, pioglitazone has been associated with adverse effects, including weight gain and fluid retention, which may complicate its use in elderly patients with comorbid conditions [12].

With the increasing adoption of biomarker-supported AD diagnosis through cerebrospinal fluid (CSF) markers and amyloid PET imaging, variability in clinical trial methodologies remains a challenge. While some studies rely exclusively on standardized cognitive assessments (e.g., MMSE), others incorporate biomarker-based confirmation. To provide a comprehensive evaluation, our analysis considers both cognitive and biomarker-based studies where available.

A critical gap in the current literature is the absence of head-to-head comparisons between different antidiabetic agents for AD. Most studies have compared individual drugs to placebo, leaving unresolved questions about their relative efficacy and safety. To address these gaps, a robust network meta-analysis is needed to integrate available evidence, compare the efficacy and safety profiles of various antidiabetic agents, and provide a systematic evaluation that can inform both future research and clinical practice.

## 2 Methods

### 2.1 Study design

This study is a systematic review and network meta-analysis designed to evaluate the efficacy, safety, and tolerability of antidiabetic agents in AD. The study was conducted in adherence to the Preferred Reporting Items for Systematic Reviews and Meta-Analyses (PRISMA) guidelines to ensure transparency and methodological rigor. RCTs were exclusively included in the analysis to provide high-quality evidence, minimize bias, and strengthen the validity of the findings.

### 2.2 Data sources and search strategy

A comprehensive literature search was conducted across several electronic databases, including PubMed, Embase, the Cochrane Library, and Web of Science. The search strategy utilized Medical Subject Headings (MeSH) terms, keywords, and Boolean operators to identify relevant studies. Key terms included combinations of condition-specific terms (e.g., "Alzheimer's disease," "mild cognitive impairment," "dementia"), intervention terms (e.g., "antidiabetic agents," "insulin," "liraglutide," "exenatide," "metformin," "pioglitazone," "GLP-1 receptor agonists," "thiazolidinediones"), and outcome-related terms (e.g., "cognition," "cognitive performance," "amyloid-β," "CSF biomarkers," "adverse effects"). A representative search string for PubMed, for instance, included: (“Alzheimer's disease” OR “dementia”) AND (“antidiabetic agents” OR “insulin” OR “GLP-1 receptor agonists” OR “metformin” OR “pioglitazone”) AND (“cognition” OR “amyloid-beta” OR “adverse effects”). The search was performed for all studies published up to November 2024, with no language restrictions applied to ensure broad inclusivity and data comprehensiveness.

### 2.3 Study selection criteria

Studies were included if they were RCTs examining the effects of antidiabetic agents in patients diagnosed with AD. Eligible interventions included any antidiabetic agents, such as insulin analogs, GLP-1 receptor agonists (e.g., liraglutide, exenatide), metformin, and pioglitazone. The outcomes of interest included cognitive performance, assessed using standardized tools like the Mini-Mental State Examination (MMSE), Alzheimer's Disease Assessment Scale-Cognitive Subscale (ADAS-Cog), or other validated instruments; Aβdeposition, measured using cerebrospinal fluid (CSF) biomarkers or positron emission tomography (PET) imaging; and safety or adverse effects, including the incidence of treatment-emergent adverse events, tolerability, or withdrawals due to adverse events. Studies were excluded if they were non-randomized, observational, or case reports; if they lacked relevant outcome data or did not provide sufficient quantitative information for inclusion in the network meta-analysis; if they focused on populations outside of AD (e.g., diabetes-only cohorts without cognitive assessments); or if they were unavailable in full text or could not be translated or extracted for data analysis.

This study focused on insulin analogs, GLP-1 receptor agonists, metformin, and thiazolidinediones due to their well-established role in metabolic regulation and their extensive evaluation in RCTs assessing cognitive and biomarker outcomes in AD. Although other antidiabetic agents such as DPP-4 inhibitors, sulphonylureas, and SGLT-2 inhibitors have been explored in observational studies and preclinical models, the available randomized controlled trials for these agents were either insufficient in number or lacked the necessary outcome measures for inclusion in our network meta-analysis [[13], [14], [15]]. Future studies may further explore the role of these additional classes of antidiabetic drugs in AD prevention and treatment.

### 2.4 Data extraction and quality assessment

A standardized data extraction process was implemented to ensure consistency and accuracy throughout the study. Two independent reviewers extracted relevant data from each included study using a pre-designed data extraction form. The extracted information encompassed study characteristics (e.g., author details, publication year, sample size, and study duration), intervention details (e.g., type of antidiabetic agent, dosage, route of administration, and control or comparator group), baseline characteristics (e.g., participant demographics, AD diagnosis criteria, baseline cognitive performance, and other clinical details), and key outcomes (e.g., cognitive performance via tools like MMSE or ADAS-Cog, Aβ deposition via CSF or imaging biomarkers, and safety/adverse effects such as withdrawal rates or treatment-emergent events). To ensure consistency, all extracted data were cross-checked and recorded in a structured database. The quality of the included studies was evaluated using the Cochrane Risk of Bias Tool, which assessed the risk of bias across several domains: randomization process (adequacy of random sequence generation and allocation concealment), blinding (assessment of participant, personnel, and outcome assessor blinding), incomplete outcome data (handling of missing data and attrition), selective reporting (completeness of reported outcomes), and other potential sources of bias. Each domain was rated as low, high, or unclear risk of bias, and an overall risk of bias was determined for each study.

### 2.5 Outcome measures

Cognitive function was evaluated using validated tools such as the MMSE and the ADAS-Cog. Aβ deposition was assessed through CSF biomarkers, such as Aβ42 levels, or through PET imaging, which reflect changes in the neuropathological hallmark of AD. Adverse events were recorded in terms of frequency, severity, and their impact on treatment adherence. Tolerability was assessed based on withdrawal rates due to treatment-emergent adverse events (TEAEs), which included both mild and severe AEs impacting adherence to the intervention. Additional biomarkers, such as phosphorylated tau (p-tau) levels in CSF and changes in glucose metabolism assessed via PET imaging, were included when available. Functional outcomes, including changes in activities of daily living (ADL) and global clinical assessments such as the Clinical Dementia Rating (CDR) scale, were also considered as supplementary data.

### 2.6 Statistical analysis

Statistical analysis was conducted using a frequentist framework for network meta-analysis. Data were analyzed with STATA to compare the relative efficacy, safety, and tolerability of antidiabetic agents for AD. Continuous outcomes, such as changes in cognitive performance (e.g., MMSE, ADAS-Cog), biomarkers (e.g., CSF Aβ levels), and adverse events, were analyzed using Mean Differences (MDs). For all comparisons, 95 % Confidence Intervals (CIs) were used to quantify the precision of effect estimates. Interventions were ranked using SUCRA probabilities, which assess the likelihood of an intervention being the best for a specific outcome. Statistical heterogeneity was assessed using the *I²* statistic, with values of 25 %, 50 %, and 75 % indicating low, moderate, and high heterogeneity, respectively. Heterogeneity was also evaluated visually using forest plots and quantified through τ² values, which reflect between-study variability. To assess publication bias, funnel plots were inspected for asymmetry, and statistical tests (e.g., Egger's test, Harbord's test) were applied to detect any selective reporting bias.

## 3 Results

### 3.1 Study selection and characteristics of included trials

A total of 4858 records were identified through the systematic search of PubMed, Embase, Cochrane Library, and Web of Science. After the removal of 2926 duplicates, 2926 records were screened based on titles and abstracts, resulting in 33 full-text articles assessed for eligibility. Of these, 26 studies met the inclusion criteria and were included in the final analysis. The excluded articles primarily did not meet eligibility criteria due to review articles (*n* = 6), or articles not found (*n* = 1). The PRISMA flow diagram (Figure S1) illustrates the detailed study selection process.

Table 1 summarizes the characteristics of the 26 included RCTs, comprising a total of 7361 participants. The mean age of participants ranged from 60 to 81 years, with the proportion of male participants varying from 30 % to 75 % across studies. The interventions included insulin analogs (low- and high-dose insulin detemir), GLP-1 receptor agonists such as liraglutide and exenatide, metformin, and pioglitazone. Comparators were primarily placebos, although some trials compared different dosages of the same agent. Study durations varied between 21 days and 24 months. All studies assessed cognitive performance using tools such as the MMSE or the ADAS-Cog. Aβ deposition was measured using CSF biomarkers and PET imaging. Adverse events and safety profiles were consistently reported across all studies, providing robust data for the evaluation of tolerability.

**Table 1:** Characteristics of all studies and included arms.

Author (year)	Country	Design (arm)	Sample of study (n)	Intervention		Age	Sex (% M)	Outcomes	Diagnostic criteria	AD stage of participants
				Category	Duration (Months)					

Michael Gejl (2016)	Denmark	2	38	Placebo group: Saline. Liraglutide group: Subcutaneous doses of 0.6 mg (Week 1), 1.2 mg (Week 2), and 1.8 mg (Week 3 onward).	6 Months	Placebo group: 66.6 (50–80; 1.8); Liraglutide group: 63.1 (55–70; 1.3).	Placebo group: 75 %; Liraglutide group: 42.8 %.	Ab deposition; Glucose metabolic rate measured; WMS-IV; Fasting plasma-glucose; Glycated hemoglobin; Plasma-cholesterol total; Body Mass Index	MMSE, CDR score	Wechsler Memory Scale: Placebo group: 27.2 (0–57; 3.8); Liraglutide group: 27.1 (5–44; 3.4)
Kathleen T. Watson (2018)	USA	2	43	Placebo group: Saline in similar volumes. Liraglutide group: Subcutaneous doses of 0.6 mg (Week 1), 1.2 mg (Week 2), and 1.8 mg (Week 3 onward).	3 Months	Placebo group: 59.56 (5.69); Liraglutide group: 60.88 (5.79).	Placebo group:31 %; Liraglutide group: 44 %.	BNT; BVRT total; d-KEFS; CVLT-II L; RCFT; WAIS-III	MMSE, ADAS-Cog score	BNT: Placebo group: 57.57±2.44; Liraglutide group: 57.00±3.08
Roger J. Mullins (2019)	USA	2	21	Placebo group: Saline. Exenatide group: 5 mg twice daily for 1 week, then increased to 10 mg twice daily.	18 Months	Placebo group: 74.0 ± 6.4; Exenatide group: 71.7 ± 6.9.	Placebo group:40 %; Exenatide group: 64 %.	FAB total; ADAS-Cog; MMSE total; CDR global; CDR sob; Verbal Fluency; CVLT Trials; CVLT SDFR; CVLT LDFR; CVLT RE; Boston SC; Boston FIF; BMI; Fasting Glucose	MMSE, CDR score	MMSE score: Placebo group: 26.0 ± 3.5; Exenatide group: 25.5 ± 4.2
Amy Claxton (2015)	USA	3	60	Placebo group: Saline. Low Dose group: 20 IU insulin detemir daily (10 IU b.i.d.). High Dose group: 40 IU insulin detemir daily (20 IU b.i.d.).	21 days	Placebo group: 71.45 (1.87); Low Dose group: 71.76 (1.88); High Dose group: 72.74 (1.93).	Placebo group: 65.0 %; Low Dose group:61.9 %; High Dose group: 57.9 %.	APOE 4 carriage interaction; Verbal working memory; BMI; HOMA-IR; Adverse events	MMSE score	MMSE score: Placebo group: 25.60 ± 0.98; Low Dose group: 26.33 ± 0.88
Suzanne Craft (2012)	USA	3	104	Placebo group: Saline. Low Dose group: 20 IU insulin detemir daily (10 IU twice daily). High Dose group: 40 IU insulin detemir daily (20 IU twice daily).	4 months	Placebo group: 74.9 (1.6); Low Dose group: 72.8 (1.5); High Dose group: 69.9 (1.4).	Placebo group: 56.7 %; Low Dose group:61.1 %; High Dose group: 52.6 %.	Delayed story recall score; DSRS score; ADAS-cog score; ADCS-ADL scale score	ADAS-Cog score, aMCI score	ADAS-Cog score: Placebo group: 11.98±3.13; Low Dose group: 12.21±3.12
Suzanne Craft (2017)	USA	3	36	Placebo group: Saline. Insulin detemir group: 40 IU daily for four months. Regular insulin group: 40 IU daily for four months.	4 months	Placebo group: 68.4 (8.9); Insulin detemir group: 70.5 (9.1); Regular insulin group: 67.3 (7.8).	Placebo group: 50 %; insulin detemir group: 41.6 %; Regular insulin group: 50 %.	Memory composite; Global cognition; Ddaily functioning; MRI volume changes; Cerebrospinal fluid AD markers	MMSE score	MMSE score: Placebo group: 25.8 ± 4.2; insulin detemir group: 26.0 ± 3.7
Suzanne Craft (2020)	USA	3	240	Placebo group: Saline. Regular Insulin group: 40 IU. Detemir Insulin group: 40 IU.	12 Months	Placebo group: 71.1 (6.8); Insulin group: 70.5 (7.4).	Placebo group: 51.3 %; Insulin group: 51.2 %.	ADAS-cog-12; Safety and adherence	MMSE score	MMSE score: Placebo group: 24.84 ± 2.72; insulin group: 24.79 ± 2.75
José A. Luchsinger (2016)	USA	2	80	Placebo group: Matching placebo from Merck-Lipha, France. Metformin group: 500 mg once daily, titrated weekly to 1000 mg twice daily over 4 weeks.	12 Months	Placebo group: 64.1 (7.9); Metformin group: 65.3 (7.0).	Placebo group: 55 %; Metformin group: 40 %.	ADAS-cog; WMS-R; rCMRgl; Plasma Aβ42	Physical exam, blood tests, and a neuropsychological battery	ADAS-Cog score: Placebo group:12.0 ± 4.0; Metformin group:14.6 ± 6.1
Aaron M. Koenig (2017)	USA	2	20	Placebo group: Matching placebo. Metformin group: 500 mg daily for 1 week, then increased by 500 mg weekly until reaching 2000 mg/day (1000 mg twice daily).	4 Months	Placebo group: 71.1 (6.57); Metformin group: 69.1 (7.40).	Placebo group: 60 %; Metformin group: 50 %.	Safety; CSF; Cognition	Physical exam, blood tests, and a neuropsychological battery	MMSE score: Placebo group:26.9 ± 1.46; Metformin group: 25 ± 2.55
Tomohiko Sato (2011)	Japan	2	42	Placebo group: Matching placebo. Pioglitazone group: 15–30 mg daily.	6 Months	Placebo group: 77.6 ± 6.5; Pioglitazone group: 77.4 ± 6.2.	Placebo group: 52.3 %; Pioglitazone group: 42.8 %.	Metabolic; Neuropsychological; and plasma Aβ40 and Aβ42 changes; Adverse events	Alzheimer's Disease and Related Disorders Association criteria	MMSE score: Placebo group:22.1 ± 3.5; Pioglitazone group:21.9 ± 3.4
G. Stennis Watson (2005)	USA	2	30	Placebo group: Matching placebo. Pioglitazone group: 4 mg daily oral dose of rosiglitazone.	6 Months	Placebo group: 73.3 (6.0); Pioglitazone group: 72.8 (6.6).	Placebo group: 70 %; Pioglitazone group: 70 %.	Metabolic Response; Cognitive Response; Ab Response	MMSE score, a lumbar puncture, and an MRI	MMSE score: Placebo group:22.7 ± 4.5; Pioglitazone group: 23.3 ± 5.4
Jinyang Zheng (2017)	China	2	49	Control group: Donepezil (5–10 mg nightly), metformin, and/or non-sulfonylurea insulin secretagogues. GLP-1Rs group: Same as control, plus Exenatide (5–10 μg/day).	12 Months	Control group: 80 ± 10.3; GLP-1Rs group: 81 ± 10.3.	Control group: 100 %; GLP-1Rs group: 100 %.	MMSE score	MMSE score	MMSE score: Placebo group: 16.2 ± 1.61; GLP-1Rs group: 15.4 ± 1.26
Shihua Hong (2016)	China	2	60	Control group: Hypoglycemic, hypotensive, and lipid-lowering treatment. GLP-1Rs group: Same as control, plus Liraglutide injection (18 mg/3 ml, Novo Nordisk, China), starting at 0.6 mg daily at 22:00. After 7 days, increased to 1.2 mg daily, with a maximum dose of 1.8 mg, and adjustments to other hypoglycemic medications.	6 Months	Control group: 75.91 ± 12.46; GLP-1Rs group: 72.21 ± 10.34.	Control group: 36.6 %; GLP-1Rs group: 56.7 %.	MMSE score	MMSE score	MMSE score: Placebo group: 22.5 ± 2.5; GLP-1Rs group: 22.5 ± 2.3
Weicong Zhang (2023)	China	2	117	Control group: Donepezil alone. Metformin group: Metformin combined with Donepezil.	12 Months	Control group: 69.80 ± 7.31; Metformin group: 70.00 ± 7.08.	Control group: 33.8 %; Metformin group: 31 %.	Safety; MMSE score	MMSE score	MMSE score: Placebo group: 17.89±3.67; Metformin group: 16.38±4.55
Yanjun Guo (2017)	China	2	120	Control group: Conventional diabetes medication. Metformin group: 500 mg metformin hydrochloride tablets (Jiangsu Suzhong Pharmaceutical), three times daily in addition to control treatment.	6 Months	Control group: 66.88 ± 4.37; Metformin group: 66.39 ± 4.70.	Control group: 30 %; Metformin group: 30 %.	Aβ deposits	MMSE, ADAS-Cog score	MMSE score: Placebo group:21.62±3.57; Metformin group: 21.36±3.38
Michael Gold (2010)	UK	4	553	Placebo group: Matching placebo. Pioglitazone group: Rosiglitazone 2 mg, 8 mg, or 10 mg.	6 Months	Placebo group: 72.5 ± 8.56; Rosiglitazone (2 mg): 71.7 ± 7.91; Rosiglitazone (8 mg): 72.6 ± 8.63; Rosiglitazone (10 mg): 72.9 ± 7.97.	Placebo group: 40 %; Rosiglitazone (2 mg): 36 %; Rosiglitazone (8 mg): 35 %; Rosiglitazone (10 mg): 37 %.	ADAS-Cog; Safety	MMSE score	MMSE score: Placebo group: 19.68±4.04; Rosiglitazone group: 19.48±4.01
Sofia Tzimopoulou (2010)	UK	2	76	Placebo group: Matching placebo. Pioglitazone group: RSGXR 4 mg daily for the first month, increasing to 8 mg daily for the remaining 11 months.	12 Months	Placebo group: 69.9 (52, 84); Pioglitazone group: 72.2 (53, 85).	Placebo group: 52.6 %; Pioglitazone group: 55.3 %.	MMSE scores; [18F]FDG uptake; CMRglc	MMSE score	MMSE score: Placebo group: 21.33±4.12; Rosiglitazone group: 21.45±4.26
C. Harrington (2011)	USA	3	1393	Placebo group: Matching placebo. Pioglitazone group: Rosiglitazone 2 mg or 8 mg.	12 Months	Placebo group: 74.0 (50–90); Rosiglitazone (2 mg): 74.2 (50–90); Rosiglitazone (8 mg): 74.1 (50–91).	Placebo group: 49 %; Rosiglitazone (2 mg): 43 %; Rosiglitazone (8 mg): 39 %.	Adverse Events; ADAS-Cog	MMSE score	MMSE score: Placebo group: 19.6 ± 4.1; Rosiglitazone group:19.5 ± 4.0
D. Kellar (2021)	USA	2	40	Placebo group: Saline. Insulin detemir group: 20 IU daily for four months.	12 Months	Placebo group: 70.88 (5.69); Insulin detemir group: 71.69 (8.25).	Placebo group: 70 %; Insulin detemir group: 55 %.	Cognitive Outcomes; CSF outcomes	CDR, MMSE, ADAS-Cog13, a lumbar puncture, and an MRI	ADAS-Cog score: Placebo group:24.75 ± 8.75; Insulin detemir group:24.15 ± 7.36
M.A. Reger (2008)	USA	2	25	Placebo group: Saline. Insulin detemir group: 20 IU BID intranasal insulin.	21 days	Placebo group: 79.3 (1.7); Insulin detemir group: 77.1 (1.6).	Placebo group: 61 %; Insulin detemir group: 58 %.	Cognition; β-amyloid levels	MMSE score, ADAS-Cog score	ADAS-Cog score: Placebo group:24.13 ± 6.46; Insulin detemir group:25.11 ± 9.17
Michael Rosenbloom (2021)	USA	2	35	Placebo group: Saline. Insulin detemir group: 20 IU daily for four months.	8 Months	Placebo group: 74.4 (6.4); Insulin detemir group: 68.4 (8.1).	Placebo group: 62.5 %; Insulin detemir group: 52.6 %.	Adverse events; Cognition	MMSE score, ADAS-Cog score	ADAS-Cog score: Placebo group:23.3 ± 6.1; Insulin detemir group:23.2 ± 5.4
Michael Gejl (2017)	Denmark	2	38	Placebo group: Saline. Liraglutide group: Liraglutide administered.	6 Months	Placebo group: 74.5 (6.2); Liraglutide group: 68.8 (8.3).	Placebo group: 72.8 %; Liraglutide group: 67.3 %.	CMRglc	MMSE score	MMSE score: Placebo group: 24.1 ± 3.6; Liraglutide group: 23.8 ± 4.0
Ivan Koychev (2024)	UK	2	3973	Placebo group: Saline. Exenatide group: Subcutaneous EQW 2 mg.	24 Months	Placebo group: 62.42 (9.30); Exenatide group: 61.99 (9.48).	Placebo group: 60 %; Exenatide group: 61 %.	Cognition	MMSE score	MMSE score: Placebo group: 25.5 ± 3.8; Exenatide group: 25.1 ± 4.1
A. Dei Cas (2024)	Italy	2	32	Placebo group: Saline. Exenatide group: 2 mg once-weekly subcutaneous injection.	8 Months	Placebo group: 72 ± 6; Exenatide group: 74 ± 4.	Placebo group: 53 %; Exenatide group: 47 %.	ADAS-Cog score; Adverse events	MMSE score	MMSE score: Placebo group: 26.1 ± 1.8; Exenatide group: 25.9 ± 1.3
Haruo Hanyu (2009)	Japan	2	32	Placebo group: Matching placebo. Pioglitazone group: 15–30 mg daily.	6 Months	Placebo group: 76.5 ± 5.8; Pioglitazone group: 77.9 ± 6.2.	Placebo group: 53.3 %; Pioglitazone group: 47 %.	Cognition	MMSE score	Alzheimer's Disease Assessment Scale Cognitive subscale: Placebo group: 15.8 ± 6.0; Pioglitazone group: 15.6 ± 4.9
Massimiliano Plastino (2010)	Italy	2	104	Control group: Oral antidiabetic medication. Insulin group: Insulin plus oral antidiabetic medications.	12 Months	Control group: 73.7 (10.2); Insulin group: 81.7 (8.2).	Control group: 46.9 %; Insulin group: 47.2 %.	Neuropsychological assessment	MMSE score	MMSE score: Placebo group: 21.9 ± 5.1; insulin group: 20.4 ± 4.1

**Fig. 1:**
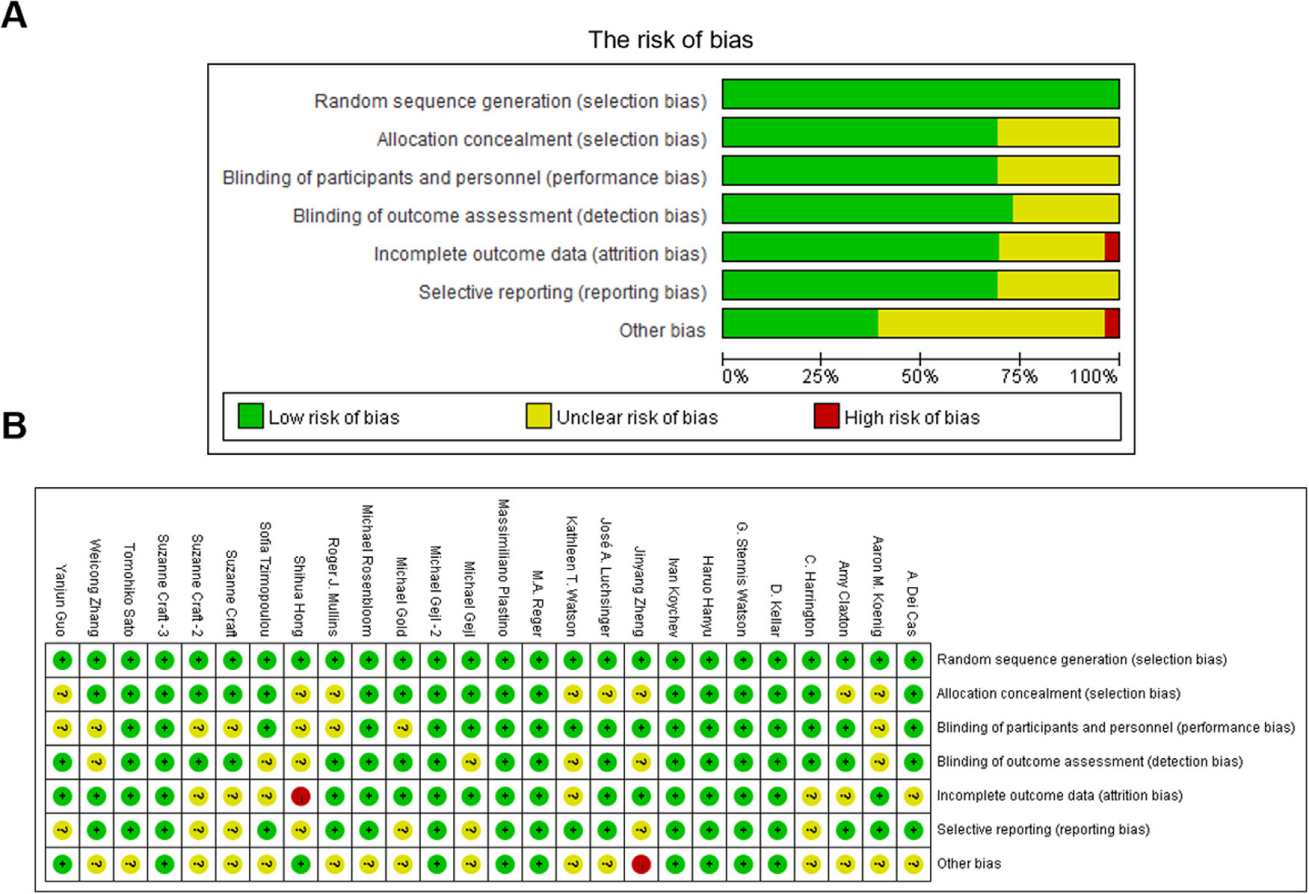
The risk of bias summary for studies included in the meta-analysis.

The included studies were generally of moderate to high quality. Most studies demonstrated a low risk of bias in random sequence generation and selective reporting. However, concerns were noted in areas such as allocation concealment and blinding of participants and outcome assessors, where several studies showed unclear or high risks of bias. Overall, the quality of evidence supports the reliability of the findings, though certain limitations should be considered (Fig. 1).

### 3.2 SUCRA rankings

**Fig. 2:**
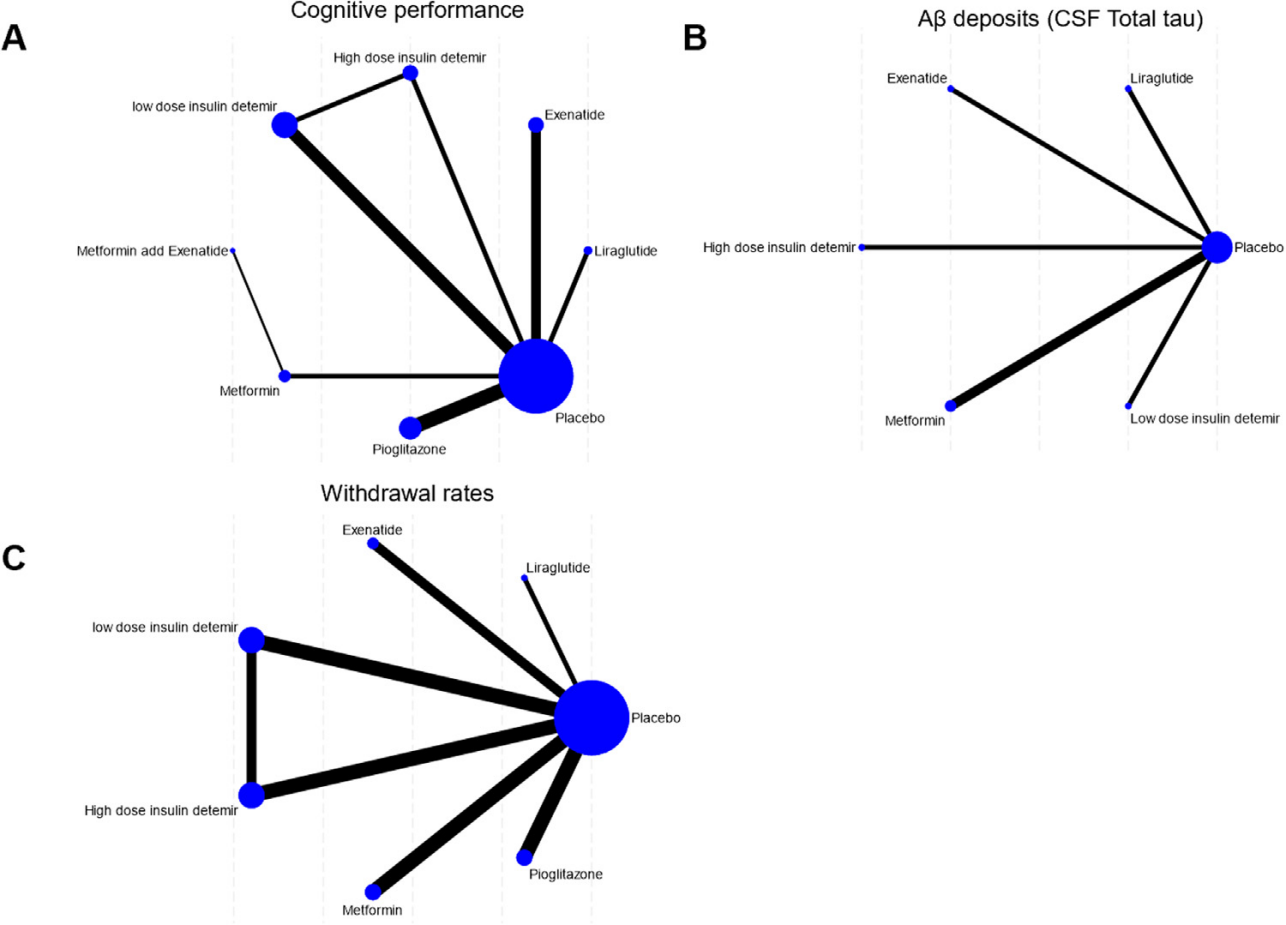
Network. (A) Cognitive performance. (B) Aβ deposits (CSF Total tau). (C) Withdrawal rates.

**Fig. 3:**
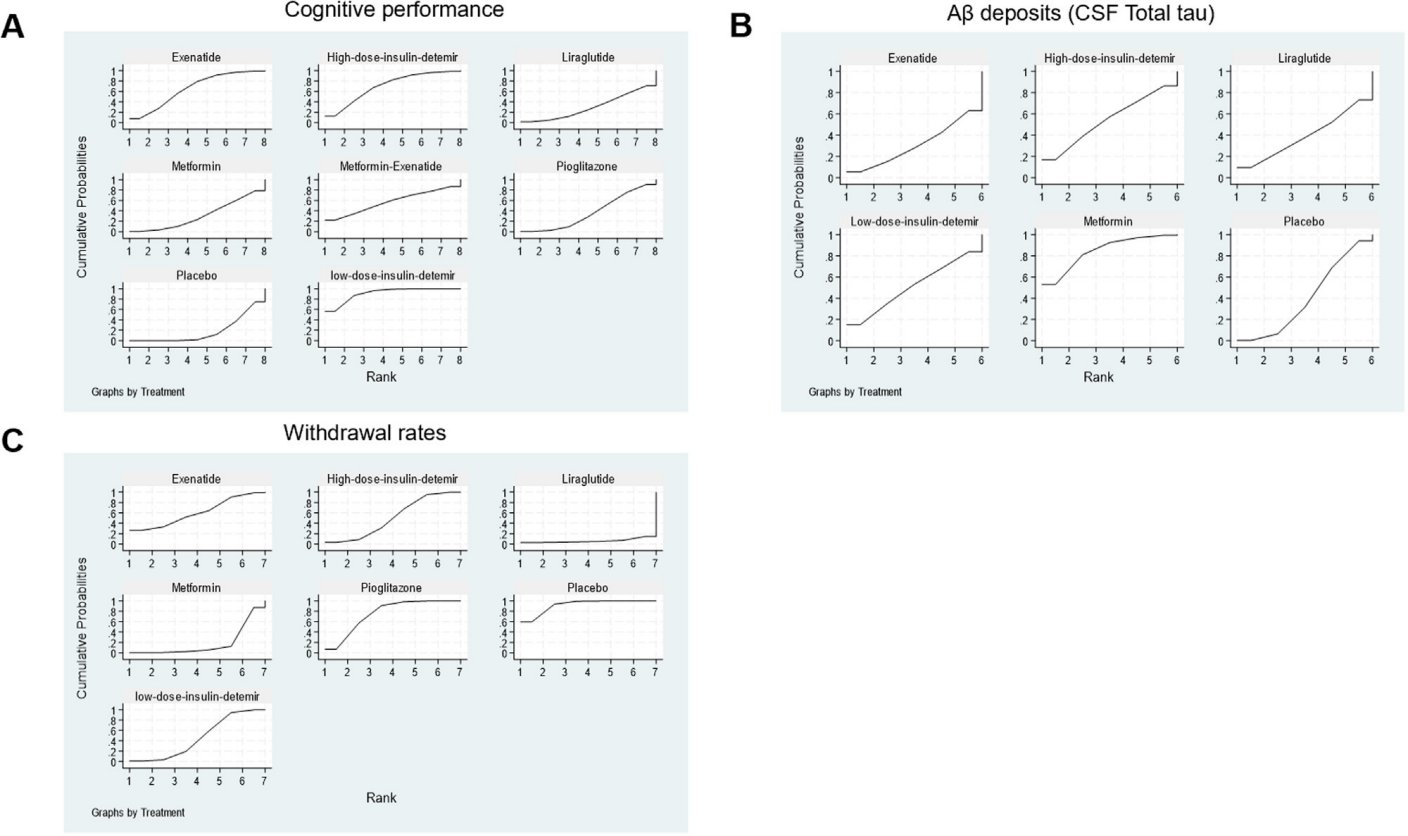
SUCRA plot for (A) Cognitive performance. (B) Aβ deposits (CSF Total tau). (C) Withdrawal rates.

**Fig. 4:**
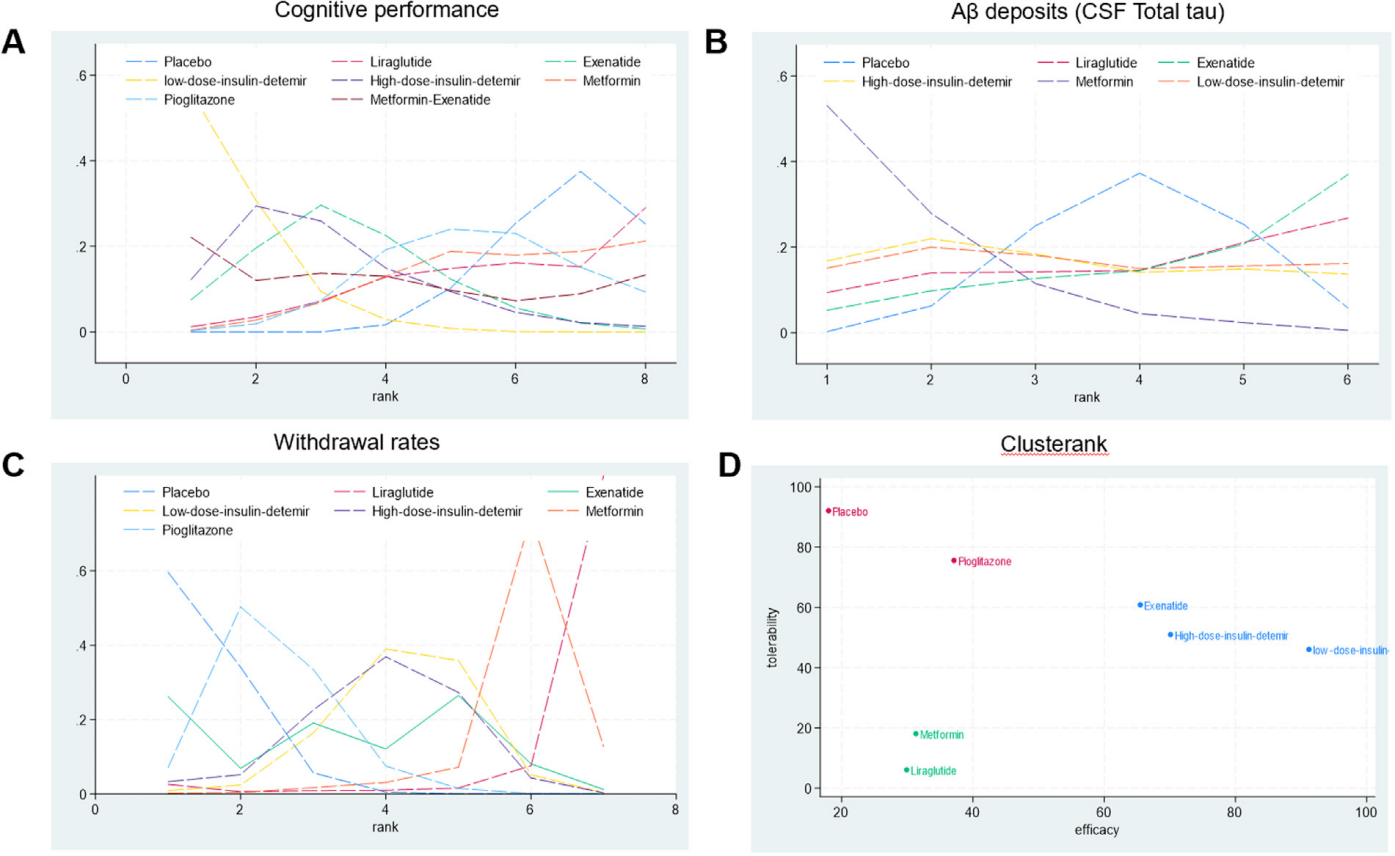
Rankogram for (A) Cognitive performance. (B) Aβ deposits (CSF Total tau). (C) Withdrawal rates. Clusterank for (D).

The network structure (Fig. 2) demonstrates that most treatments were directly or indirectly compared to placebo. The network meta-analysis revealed significant differences in cognitive performance among the included antidiabetic agents. Low-dose insulin detemir ranked the highest in improving cognitive performance with a SUCRA value of 91.2 %, indicating its superiority among interventions (Fig. 3A, Supplemental Online Content). In contrast, placebo consistently ranked the lowest, as shown in the rankogram (Fig. 4A). In terms of Aβ deposition reduction, no treatment achieved statistical significance compared to placebo. However, metformin exhibited the most promising trend, with a SUCRA value of 84.6 % (Fig. 3B, Supplemental Online Content). Rankograms (Fig. 4B) further confirmed that placebo consistently ranked as one of the least effective options. Safety analyses based on study treatment withdrawal due to adverse events indicated varying profiles among the interventions. Pioglitazone had the most favorable safety profile, as indicated by a SUCRA value of 75.6 % (Fig. 3C, Supplemental Online Content), followed by Exenatide with 60.9 %. Liraglutide was associated with the highest withdrawal rates (SUCRA 6.1 %), as shown in Fig. 4C. Placebo ranked highly in safety but lacked efficacy, as evident in the cluster rank diagram (Fig. 4D), which juxtaposes efficacy and tolerability for all interventions.

### 3.3 Network meta-analysis

**Fig. 5:**
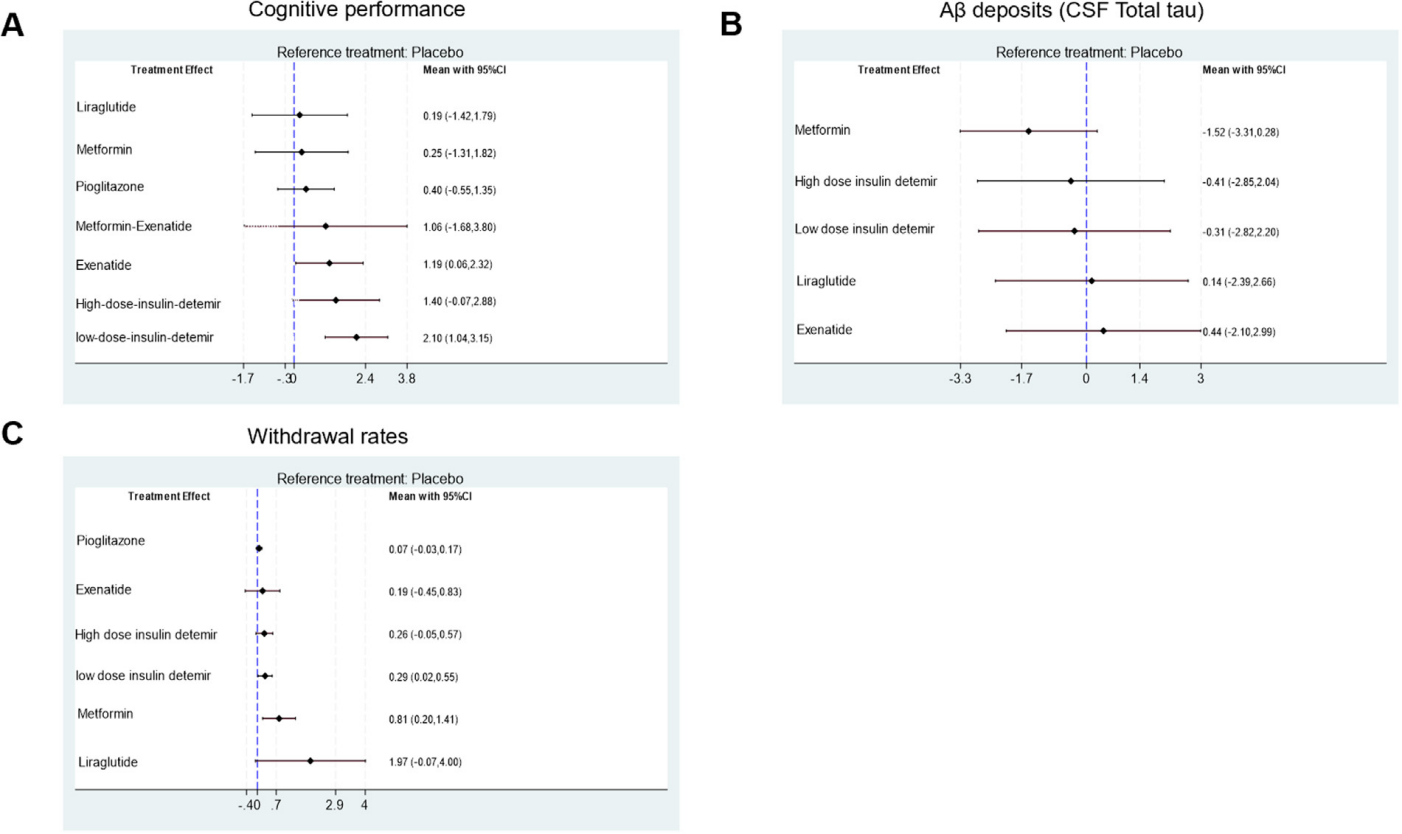
Forest plot for (A) Cognitive performance. (B) Aβ deposits (CSF Total tau). (C) Withdrawal rates.

**Fig. 6:**
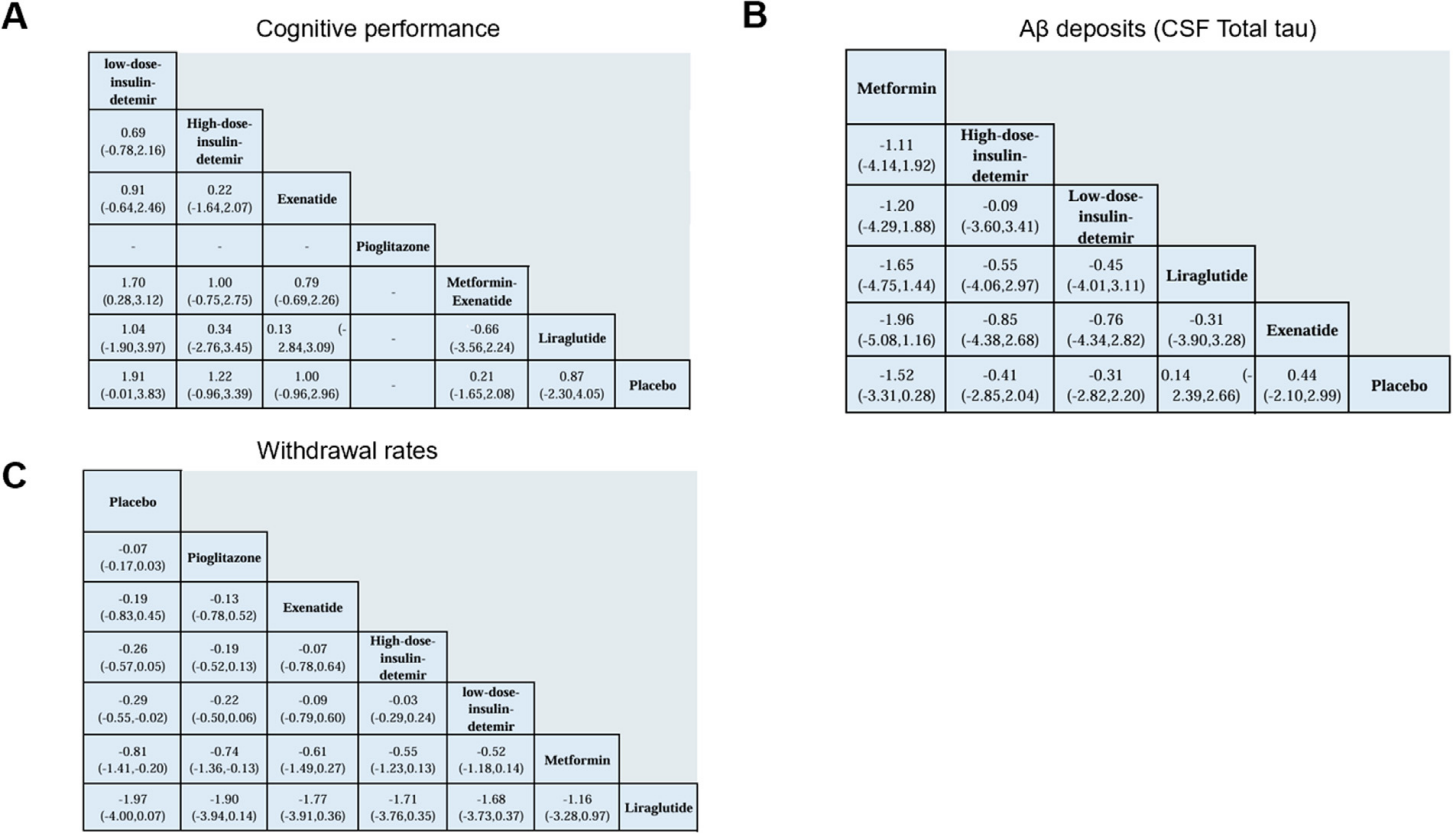
Network league of outcomes (A) Cognitive performance. (B) Aβ deposits (CSF Total tau). (C) Withdrawal rates.

The analysis highlights differences in cognitive outcomes among antidiabetic agents. Low-dose insulin detemir demonstrated the strongest positive effect on cognitive performance, with a significant mean difference (MD = 2.10, 95 % CI: 1.04 to 3.15). Exenatide also showed notable efficacy (MD = 1.19, 95 % CI: 0.06 to 2.32). metformin-Exenatide provided modest improvements (MD = 1.06, 95 % CI: −1.68 to 3.80), while Liraglutide, metformin and pioglitazone exhibited minimal or non-significant effects (Fig. 5A, Fig. 6A).

The effect on Aβ deposits revealed varied results. Metformin showed a reduction in tau levels (MD = −1.52, 95 % CI: −3.31 to 0.28), though confidence intervals crossed null, indicating inconsistency. Insulin detemir at both high and low doses exhibited modest reductions in tau deposition. Liraglutide and exenatide demonstrated smaller but similar trends of reduction, while placebo offered no benefit (Fig. 5B, Fig. 6B).

Safety profiles varied among interventions. Liraglutide exhibited the highest rate of study treatment withdrawal, with a MD of 1.97 (95 % CI: 0.07 to 4.00) compared to placebo, representing an absolute difference in the proportion of withdrawals. In contrast, pioglitazone was associated with the lowest withdrawal rates, with an MD of 0.07 (95 % CI: −0.03 to 0.17). Other agents, such as insulin detemir and metformin, demonstrated acceptable safety profiles, with withdrawal rates remaining within a tolerable range (Fig. 5C, Fig. 6C).

### 3.4 Publication bias

Publication bias was assessed using funnel plots. The funnel plots for cognitive performance, Aβ deposition outcomes, and withdrawal rates were visually symmetrical, suggesting no significant publication bias. These analyses underscore the reliability and robustness of the included studies and the synthesized evidence (Fig. S2).

## 4 Discussion

The main findings of this study indicate significant differences in cognitive performance and safety profiles among various antidiabetic agents. Low-dose insulin detemir showed the greatest improvement in cognitive performance, outperforming other treatments, while placebo consistently ranked lowest. Although no treatment demonstrated statistically significant effects on Aβ deposition compared to placebo, metformin showed the most promising trend. In terms of safety, pioglitazone had the most favorable profile, while liraglutide was associated with a highest withdrawal rates. These results offer valuable insights into the impact of antidiabetic medications on cognitive function.

### 4.1 Comparison with previous studies

Research on insulin analogs and their effects on cognitive performance has produced mixed results. In animal models, intracerebroventricular administration of insulin detemir has been shown to improve cognitive function and reduce Alzheimer's-like pathology in rats [16]. However, when compared to regular insulin, the impact of insulin detemir on human cognition appears less pronounced. In a clinical trial, regular insulin administered intranasally for four months improved memory and preserved brain volume in patients with mild cognitive impairment or AD, whereas insulin detemir showed no significant effects [17]. Furthermore, studies indicate that insulin and insulin glargine exert greater effects on central nervous system functions than insulin detemir [18]. Interestingly, during hypoglycemia, insulin detemir demonstrated higher glycemic thresholds for adrenergic symptoms and greater maximal responses to both adrenergic and neuroglycopenic symptoms compared to regular insulin, but it also caused earlier and more severe impairment of cognitive function [19].

Metformin, a commonly prescribed medication for type 2 diabetes, has shown promise in both reducing the risk of AD and improving cognitive performance. Its effects on AD pathology include modulation of Aβ deposition, tau phosphorylation, and neuroinflammation [20]. Metformin activates the AMPK pathway, suppresses mTOR, and reduces BACE-1 protein expression in animal models, suggesting a potential neuroprotective effect [21].

Pioglitazone, a thiazolidinedione used in type 2 diabetes treatment, has recently garnered attention for its favorable safety profile. While it shares some common adverse effects with other drugs in its class, such as weight gain and edema, pioglitazone has shown a reduced risk of cardiovascular events in most studies [22]. Nevertheless, it has also been associated with an increased risk of heart failure, edema, and bladder cancer, which requires careful monitoring. Long-term clinical trials and post-marketing surveillance have supported pioglitazone's overall safety, highlighting its potential for broader use in the management of diabetes [23].

### 4.2 Mechanistic implications

GLP-1 receptor agonists, initially developed for type 2 diabetes, have shown promising neuroprotective effects in AD models. These drugs improve learning and memory in AD rodents, while also reducing Aβ deposition and tau phosphorylation [7]. Their mechanisms of neuroprotection include the reduction of neuroinflammation, oxidative stress, and the enhancement of neurotrophic effects [24]. Additionally, GLP-1 mimetics decrease beta-amyloid plaques and mitigate inflammatory brain responses in experimental AD models [25]. They also protect synapses, improve cognition and motor function, regulate calcium homeostasis, reduce endoplasmic reticulum (ER) stress, enhance neuronal insulin sensitivity, and promote neurogenesis [26]. Given their safety profile and their ability to target multiple neuroprotective pathways, GLP-1 receptor agonists hold promise as a potential treatment for AD.

Insulin and its analogs have also demonstrated beneficial effects on brain function by interacting with neuronal insulin receptors. In cultured rat neurons, insulin, glargine, and detemir increased Akt phosphorylation and brain-derived neurotrophic factor (BDNF) expression, though detemir's effects were diminished by albumin [27]. Neuronal insulin receptors were primarily localized on neurites, and insulin was found to modulate norepinephrine uptake in rat brain cultures [28]. In vivo studies revealed that insulin detemir produced faster and more pronounced effects on insulin signaling in the hypothalamus and cerebrocortex compared to human insulin, suggesting a selective action favoring brain tissues [29]. These findings underscore the potential of insulin analogs to improve brain function in AD.

Metformin, a well-established antidiabetic medication, also shows potential neuroprotective effects in AD through various mechanisms. It activates AMP-activated protein kinase (AMPK)-dependent pathways in neural stem cells, reduces the expression and activity of beta-secretase 1 (BACE1), decreases Aβ production, and lowers acetylcholinesterase activity [9]. Metformin has also been shown to improve glucose metabolism, reduce amyloid plaque deposition, normalize tau phosphorylation, and enhance autophagy [30]. Furthermore, it addresses neuronal loss, neural dysfunction, chronic neuroinflammation, insulin resistance, and mitochondrial dysfunction [31]. These multifaceted actions position metformin as a promising candidate for AD treatment.

Peroxisome proliferator-activated receptor gamma (PPARγ) agonists have emerged as another promising therapeutic approach for AD. PPARγ is a ligand-activated transcription factor that regulates glucose and lipid metabolism, while also suppressing inflammatory gene expression [32]. In AD, PPARγ agonists have been shown to improve disease-related pathology and cognitive function in animal models [33]. These effects are attributed to several mechanisms, including modulation of microglial activation, reduction of inflammatory responses, and modulation of Aβ homeostasis [34]. Pioglitazone, a PPARγ agonist, has been found to alleviate neuroinflammation by regulating microglial activation and reducing pro-inflammatory cytokine production [35]. PPARγ agonists represent a promising strategy for addressing the inflammatory and metabolic components of AD.

In conclusion, the therapeutic potential of GLP-1 receptor agonists, insulin analogs, metformin, and PPARγ agonists lies in their ability to target various pathophysiological mechanisms in AD, offering hope for more effective treatments.

### 4.3 Strengths and limitations

#### 4.3.1 Strengths

One of the key strengths of this study is the comprehensive inclusion of a wide range of antidiabetic agents, including GLP-1 receptor agonists, insulin analogs, metformin, and pioglitazone, allowing for a thorough evaluation of their relative efficacy and safety in AD. The use of a robust network meta-analysis methodology enabled the integration of both direct and indirect evidence, providing a systematic and hierarchical comparison of interventions even when head-to-head trials were unavailable. This approach ensured a comprehensive assessment of the effectiveness and tolerability of these therapies. Another notable strength is the rigorous assessment of primary outcomes, including cognitive performance and Aβ deposition, alongside secondary outcomes such as withdrawal rates. By evaluating multiple outcomes across diverse therapeutic mechanisms, the analysis offers clinically meaningful insights into the potential of antidiabetic agents to address the multifactorial pathology of AD. Additionally, the use of SUCRA probabilities allowed for an evidence-based ranking of interventions, enhancing the clinical applicability of the findings.

#### 4.3.2 Limitations

Despite its strengths, this study has several limitations that warrant consideration. First, there was heterogeneity in the design, duration, and population characteristics of the included trials. Differences in disease stage, baseline cognitive performance, and comorbidities among study populations may have influenced the observed treatment effects, limiting the generalizability of the findings to all AD populations. Second, the analysis relied on indirect comparisons for some interventions due to the lack of sufficient head-to-head trials. Although the network meta-analysis methodology accounts for such limitations, the reliance on indirect evidence introduces potential biases and reduces the precision of some estimates. Additionally, unmeasured confounders, such as variations in concomitant therapies or adherence rates, could not be fully accounted for and may have influenced the results. Third, there was variability in the reporting of biomarkers and cognitive outcomes across trials. While most studies used standardized measures such as MMSE or ADAS-Cog, differences in reporting intervals and analytical methods could have contributed to inconsistencies in the pooled results. Similarly, the heterogeneity in biomarker assessments, including variations in CSF collection protocols and imaging modalities, complicates the interpretation of Aβ deposition outcomes. Finally, while sex differences were evaluated in our analysis, ethnicity was not consistently reported across the included studies. Given the well-established genetic and environmental influences on AD risk among different ethnic groups, future research should prioritize more diverse trial recruitment to improve the generalizability of findings. These limitations highlight the need for further high-quality, head-to-head randomized controlled trials with standardized outcome reporting to validate these findings and refine clinical recommendations.

## 5 Conclusion

In summary, this network meta-analysis offers valuable insights into the comparative effectiveness and safety of antidiabetic drugs in the context of AD. Low-dose insulin detemir was found to have the most significant impact on cognitive improvement, alongside a moderate reduction in Aβ deposition. Metformin, while showing the greatest reduction in Aβ levels, had a more limited effect on cognitive function. The safety profiles of these agents varied; liraglutide was linked to the highest withdrawal rates, whereas pioglitazone demonstrated the lowest withdrawal rates. These results suggest that antidiabetic agents, especially insulin detemir, could be a promising option for AD treatment. However, additional studies are required to fully assess their long-term safety and effectiveness.

## Ethics approval and consent to participate

This study is a meta-analysis based on publicly available data, and does not involve direct interaction with or data collection from human participants. Therefore, ethics approval is not required for this type of study.

## Consent for publication

All authors involved in this research are aware of and agree with the content of the study, and have consented to the publication of the results.

## Funding

This work was supported by multiple grants, including: The 10.13039/501100001809National Natural Science Foundation of China (Nos. 82430029, 82330025, 82370807, 82070807), The Leading Talents Program of Hunan Province (No. 2022RC3078), The 10.13039/501100004735Natural Science Foundation of Hunan Province, China (No. 2021JJ30976), The Postdoctoral Fellowship Program of the China Postdoctoral Science Foundation (Grant No. GZB20240865), The Hunan Provincial Key Laboratory of Pediatric Orthopedics (No. 2023TP1019), The Science and Technology Project of Furong Laboratory (No. 2023SK2111).

## CRediT authorship contribution statement

**Zixin Cai:** Writing – review & editing, Writing – original draft, Methodology, Investigation, Formal analysis, Data curation, Conceptualization. **Jiaxin Zhong:** Methodology, Investigation. **Guanghui Zhu:** Writing – review & editing, Supervision. **Jingjing Zhang:** Visualization, Validation, Funding acquisition.

## Declaration of competing interest

The authors declare the following financial interests/personal relationships which may be considered as potential competing interests:

Jingjing Zhang reports financial support was provided by National Natural Science Foundation of China. If there are other authors, they declare that they have no known competing financial interests or personal relationships that could have appeared to influence the work reported in this paper.

## Appendix Supplementary materials

Image, application 1
