# Supplementary material for: Comparative efficacy and safety of antidiabetic agents in Alzheimer's disease: A network meta-analysis of randomized controlled trials
Source: J Prev Alzheimers Dis. 2025 Feb 28;12(5):100111. doi: 10.1016/j.tjpad.2025.100111 (PMC12183985; doi:10.1016/j.tjpad.2025.100111)
Supplement: Supplementary file 1 [file mmc1.docx]

**Supplemental Online Content**

**Comparative Efficacy and Safety of Antidiabetic Agents in Alzheimer's Disease: A Network Meta-Analysis of Randomized Controlled Trials**

1. Fig.S1. Study Identification and Selection.
2. Fig.S2. Funnel plot. (A) Cognitive performance. (B) Aβ deposits (CSF Total tau). (C) Withdrawal rates.
3. Effects of Antidiabetic agents on Alzheimer’s disease with cognitive performance (SUCRA).
4. Effects of Antidiabetic agents on Alzheimer’s disease with Aβ deposits (CSF Total tau) (SUCRA).
5. Effects of Antidiabetic agents on Alzheimer’s disease with Withdrawal rates (SUCRA).
6. Contribution plot of studies included in the analysis. (A) Cognitive performance. (B) Aβ deposits (CSF Total tau). (C) Withdrawal rates.
7. **Fig. S1. Study Identification and Selection.**


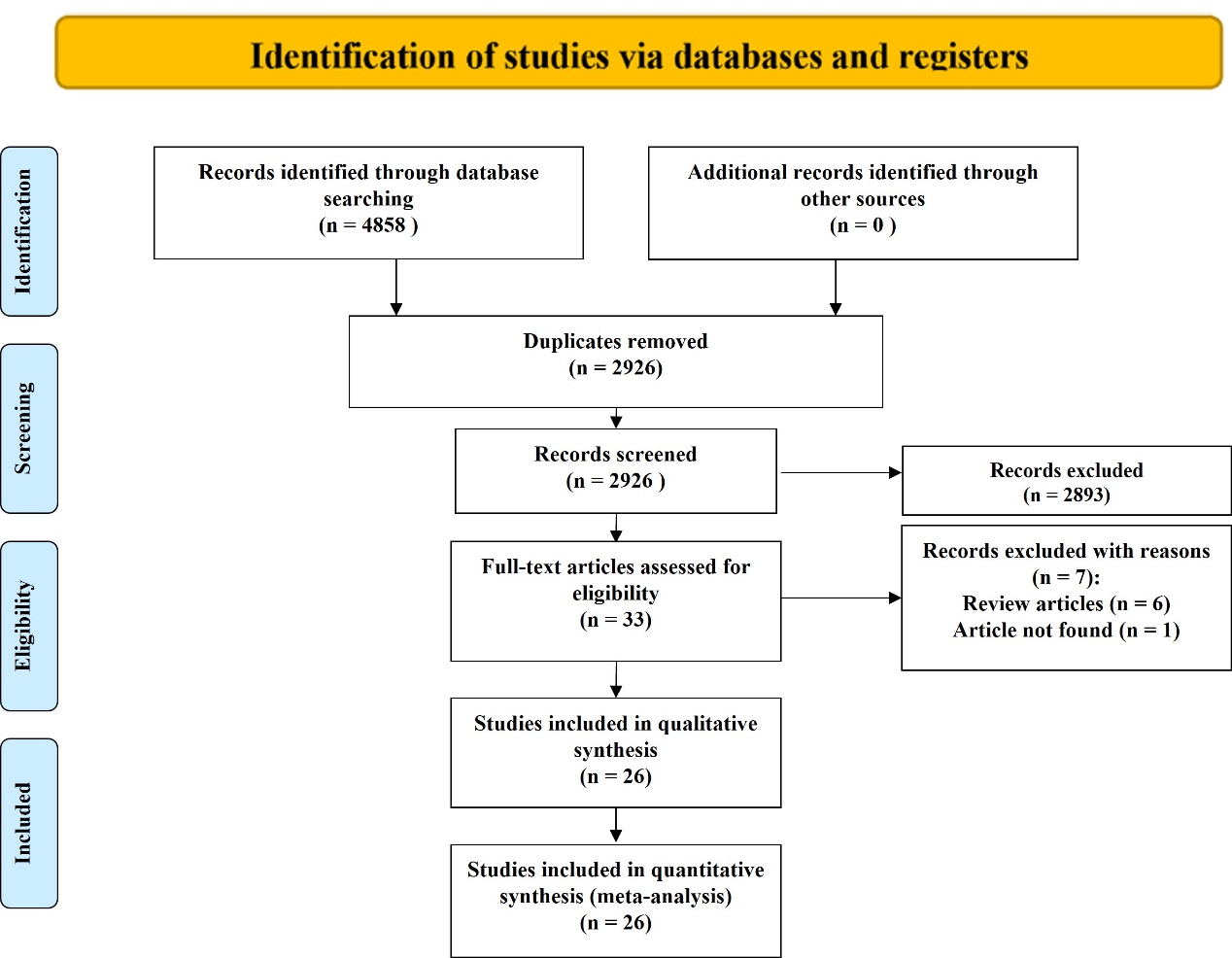


1. **Fig. S2. Funnel plot. (A) Cognitive performance. (B) Aβ deposits (CSF Total tau). (C) Withdrawal rates.**

**
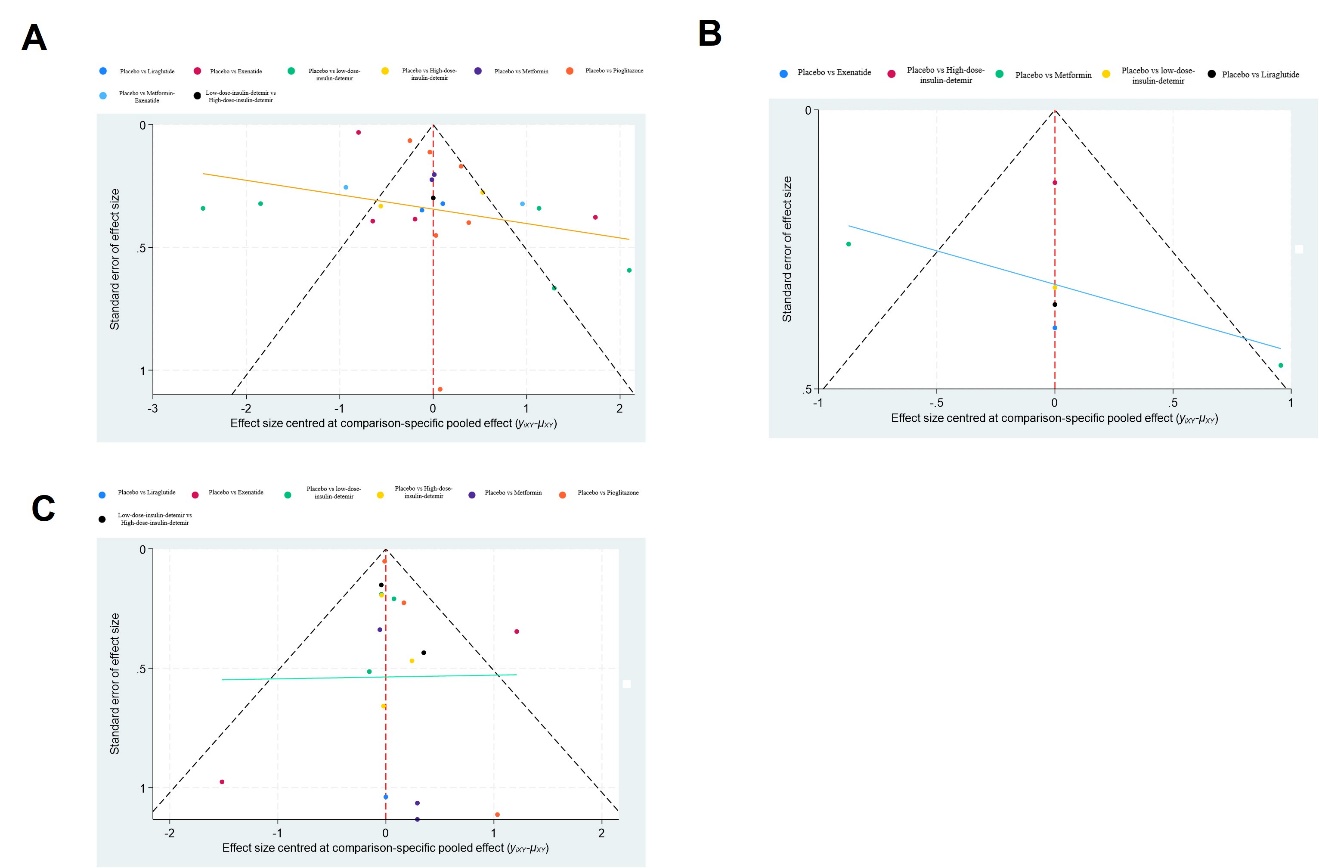
**

1. **Effects of Antidiabetic agents on Alzheimer’s disease with Cognitive performance (SUCRA).**

Treatment | SUCRA | PrBest | MeanRank |

|---------------------------+-------+--------+----------|

| Placebo | 18.0 | 0.0 | 6.7 |

| Liraglutide | 29.9 | 1.2 | 5.9 |

| Exenatide | 65.5 | 7.5 | 3.4 |

| Low-dose-insulin-detemir | 91.2 | 56.1 | 1.6 |

| High-dose-insulin-detemir | 70.1 | 12.2 | 3.1 |

| Metformin | 31.1 | 0.4 | 5.8 |

| Pioglitazone | 37.1 | 0.3 | 5.4 |

| Metformin-Exenatide | 57.1 | 22.1 | 4.0 |

1. **Effects of Antidiabetic agents on Alzheimer’s disease with Aβ deposits (CSF Total tau) (SUCRA).**

'-------------------------------------------------------+

| Treatment | SUCRA | PrBest | MeanRank |

|---------------------------+-------+--------+----------|

| Placebo | 40.3 | 0.3 | 4.0 |

| Liraglutide | 39.2 | 9.4 | 4.0 |

| Exenatide | 30.7 | 5.3 | 4.5 |

| High-dose-insulin-detemir | 54.1 | 16.8 | 3.3 |

| Metformin | 84.6 | 53.1 | 1.8 |

| Low-dose-insulin-detemir | 51.1 | 15.1 | 3.4 |

1. **Effects of Antidiabetic agents on Alzheimer’s disease with withdrawal rates (SUCRA).**

-------------------------------------------------------+

| Treatment | SUCRA | PrBest | MeanRank |

|---------------------------+-------+--------+----------|

| Placebo | 92.1 | 59.6 | 1.5 |

| Liraglutide | 6.1 | 2.6 | 6.6 |

| Exenatide | 60.9 | 26.2 | 3.3 |

| Low-dose-insulin-detemir | 46.1 | 0.9 | 4.2 |

| High-dose-insulin-detemir | 51.0 | 3.3 | 3.9 |

| Metformin | 18.1 | 0.2 | 5.9 |

| Pioglitazone | 75.6 | 7.1 | 2.5 |

+-------------------------------------------------------+

1. **Contribution plot of studies included in the analysis.**

(a) Cognitive performance. (b) Aβ deposits (CSF Total tau). (b) Withdrawal rates.

**a.**

**b.**

**c.**
